# Ferroptosis—The “Double-Edged Sword” in Cancer: Mechanisms of Tumor Suppression/Resistance and Therapeutic Manipulation

**DOI:** 10.3390/biology15010067

**Published:** 2025-12-30

**Authors:** Danielle Quaranto, Nicole R. DeSouza, Michelle Carnazza, Augustine Moscatello, Humayun K. Islam, Xiu-Min Li, Raj K. Tiwari, Jan Geliebter

**Affiliations:** 1 Department of Pathology, Microbiology, and Immunology, New York Medical College, Valhalla, NY 10595, USA; 2 Department of Research & Development, General Nutraceutical Technology, LLC, Briarcliff Manor, NY 10510, USA; 3 Department of Otolaryngology, New York Medical College, Valhalla, NY 10595, USA; 4 Department of Dermatology, New York Medical College, Valhalla, NY 10595, USA

**Keywords:** ferroptosis, cancer, iron homeostasis

## Abstract

Ferroptosis is a non-apoptotic, iron-dependent form of cell death. The characteristic accumulation of membrane lipid peroxides that occurs is controlled by several different mechanisms including redox homeostasis, the intracellular labile iron pool, and amino acid metabolism. In cancer, ferroptosis can contribute to either tumor progression or tumor suppression. When promoted in cancer cells, ferroptosis plays an innately tumor-suppressive role; however, it can negatively affect anticancer immunity, allowing for tumor growth and progression. Cells that are traditionally known to be resistant to apoptosis and other forms of cell death have demonstrated susceptibility to ferroptosis, making this form of cell death particularly useful in terms of cancer therapeutics. This narrative review will focus on the role of ferroptosis in cancer promotion or prevention and how it can be manipulated therapeutically—whether it be induced or inhibited.

## Simple Summary

Cell death is a process that can occur in cancerous cells in a variety of forms. Ferroptosis is a type of cell death that occurs when there is an overload of iron within the cell, leading to a disruption in vital components of the cellular membrane. In cancer, ferroptosis can play a “double-edged sword” role, where it can be either beneficial or detrimental. There are several factors of ferroptosis that can either promote or prevent cancer from occurring. These factors contribute to pathways that allow it to be studied and manipulated therapeutically for the benefit of treating cancer.

## 1. Introduction

Ferroptosis is an iron-dependent form of programmed cell death that is driven by the accumulation of reactive oxygen species (ROS), leading to the formation of membrane-damaging lipid peroxides [1]. This form of cell death is considered to be distinct from other forms, such as apoptosis, pyroptosis, and necroptosis, in both morphological and mechanistic ways [2]. Morphologically, cells undergoing ferroptosis exhibit several changes in their mitochondria, including smaller size, decreased cristae, and much more condensed membranes [3]. Mechanistically, ferroptosis has yet to be identified as being initiated by executioner proteins, as is seen in many other forms of cell death [4]. Overall, cells undergoing ferroptosis appear to have a reduced cell volume, with a characteristic loss of membrane integrity [5]. The overall process of ferroptosis includes the accumulation of ferrous iron, free radical production, dysfunction of the antioxidant system, and membrane-bound lipid peroxide formation [6]. The execution of ferroptosis is classified as the oxidation of phospholipids containing polyunsaturated fatty acids (PUFAs) [7]. Dixon et al. were among the first to discuss cell death differences between ferroptosis and other forms, including apoptosis. Importantly, they were able to identify that ferroptosis could not be suppressed by inhibitors of caspases or necroptosis; thus, this form of cell death is executed by lipid peroxidation and iron metabolism [8]. Yang et al. followed up on this work and further proved that the mechanism of ferroptosis is based on lipid peroxidation and not just general ROS production [9]. Based on these key findings and identifying characteristics, several biomarkers and pharmacological interventions have been developed to investigate the pathophysiology of ferroptosis in several diseases, including cancer [10].

The manipulation of ferroptosis has become an attractive area of cancer research, as both the promotion and inhibition of this form of cell death have been seen to positively affect cancer outcomes [11]. The innate induction of this process itself is seen to be both a tumor-suppressive mechanism and a tumor-promoting mechanism [6]. Cancer cells benefit from evading ferroptosis to promote their own survival, and this provides researchers with opportunities to target the ferroptosis pathway for therapeutics [12]. Some mesenchymal and dedifferentiated cells do not display susceptibility to apoptosis and other traditional therapeutics; however, there is evidence that some of these cells do respond to ferroptosis, making this form of cell death an interesting target for otherwise refractory tumors [13]. The inhibition of ferroptosis has also been seen to be beneficial for some cancer types, but it appears to be more useful at certain stages and tumor sizes. Therefore, more research must be conducted in this area for a more detailed understanding [6]. The role that ferroptosis plays in tumor biology is paradoxical [14]. For example, ferroptosis induction was seen to aid in the progression of chronic liver disease to hepatocellular carcinoma (HCC), but it restricted the progression of established HCC [15]. Thus, an extensive understanding of the dual role that ferroptosis can play in cancer can help tremendously with targeted therapeutics [6].

Ferroptotic cell death has been found to be particularly important for cancers that display dysregulations in iron metabolism, lipid remodeling, and redox imbalances. Malignancies such as pancreatic ductal adenocarcinoma, HCC, clear cell renal cell carcinoma, and triple-negative breast cancer demonstrate these dysregulations. These cancers in particular display either extreme iron dependence or occur in cells that are involved in lipid storage or metabolism and/or glutamine/cystine uptake [16]. Therefore, their ability to be targeted using ferroptosis-based therapies could be promising.

This narrative review focuses on the role of ferroptosis and its key players in both tumor-suppressive and tumor-progressive mechanisms, and how it has been shown to be a target either promoted or inhibited in various therapeutics [17].

## 2. Players in Ferroptosis

**Figure 1:**
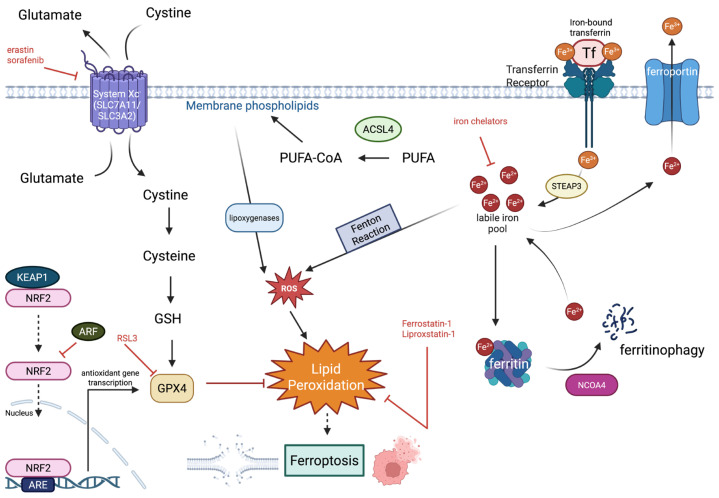
A schematic representing the signaling pathways involved in ferroptotic induction and inhibition. System Xc^−^ is a cystine/glutamate antiporter that allows for the uptake of cystine, a key precursor for glutathione (GSH) synthesis. Glutathione is an important cofactor that is used by glutathione peroxidase 4 (GPX4), which is a key antioxidant defense against lipid peroxidation. GPX4 can, in turn, be inhibited by RSL3. The KEAP–NRF2 pathway protects against lipid peroxidation and oxidative stress through the eventual transcription of antioxidant genes such as GPX4. However, ARF can inhibit NRF2, preventing transcription of antioxidant genes. Transferrin receptors can bind to iron-bound transferrin, allowing for the uptake of iron in the ferric state (Fe^3+^). Ferric iron is then converted to ferrous iron (Fe^2+^) via STEAP3, which contributes to the formation of a labile iron pool. Ferritin proteins can bind iron, but when degraded by proteins such as NCOA4, they can also release iron, contributing to the labile pool. The labile pool of iron can undergo the Fenton reaction, leading to the formation of reactive oxygen species (ROS) and peroxidation of lipids, which triggers ferroptosis. Ferroportin is a transmembrane protein that can transport iron out of the cell to reduce the labile pool and iron overload of the cell. Iron chelators can also sequester iron for a similar effect. Ferrostatin-1 and liproxstatin-1 scavenge lipid peroxides, inhibiting ferroptosis. ACSL4 activates polyunsaturated fatty acids (PUFAs) to PUFA-CoA that are needed for the synthesis of membrane phospholipids. These phospholipids can oxidize the membrane phospholipids, further contributing to the creation of ROS and lipid peroxides. [Figure created on BioRender.com].

The signaling cascades that lead to ferroptosis activation or inhibition are complex and involve many different players. There are both pro-ferroptotic and anti-ferroptotic components that either initiate or inhibit ferroptosis activation [18]; the overall pathways are represented in Figure 1. The key pathways will be discussed in this section.

### 2.1. Pro-Ferroptosis

**Figure 2:**
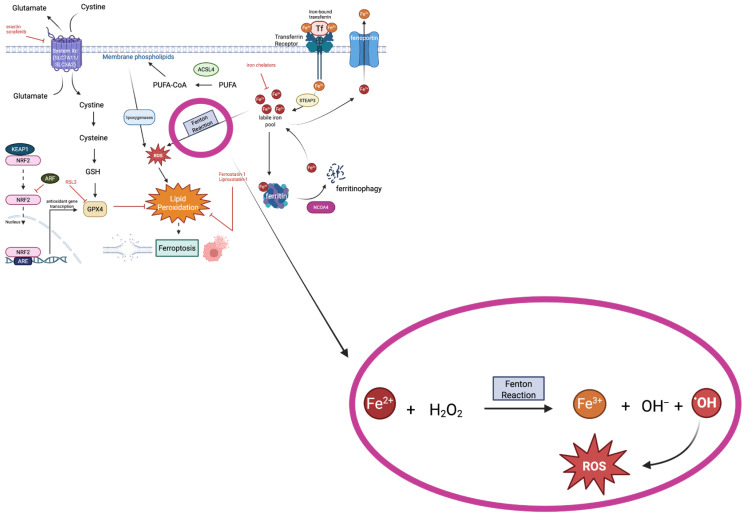
The Fenton reaction. A chemical process in which ferrous iron catalyzes the decomposition of hydrogen peroxide to produce reactive hydroxyl free radicals and ROS. The ROS produced can then lead to lipid peroxidation and subsequent ferroptosis. (This figure is adapted from Figure 1 to show the chemical process of the Fenton reaction.) [Figure created on BioRender.com].

Ferroptosis is an iron-dependent process, as iron is obligatory for this form of cell death to occur [1]. Iron in its ferrous state (Fe^2+^) is required for the Fenton reaction, a chemical process in which ferrous iron ions are used in the decomposition of hydrogen peroxide (H_2_O_2_) to create reactive hydroxyl radicals [19]; the reaction is depicted in Figure 2. Transferrin receptors on the cell surface are responsible for the uptake of ferric iron (Fe^3+^), which is then reduced to ferrous iron in the endosome through enzymes such as Six-Transmembrane Epithelial Antigen of Prostate 3 (STEAP3) metalloreductase [20]. As the amount of ferrous iron in the cell increases, a labile pool of iron is created, which can then undergo the Fenton reaction [21]. Nuclear Receptor Coactivator 4 (NCOA4) is a protein that promotes ferritin degradation, also contributing to the labile iron pool [22]. The reactive oxygen species (ROS) produced in the Fenton reaction contribute greatly to the overwhelming of the cell’s antioxidant defenses, leading to lipid peroxidation and eventual cell death [23]. Several enzymes are involved in the peroxidation of PUFAs, including Acyl-CoA Synthetase Long Chain Family Member 4 (ACSL4), Lysophosphatidylcholine Acyltransferase 3 (LPCAT3), arachidonate lipoxygenases (ALOXs), and cytochrome P450 oxidoreductase (POR) [24]. ACSL4 allows for the activation of PUFAs, making them much more susceptible to peroxidation, and LPCAT3 allows for the incorporation of PUFAs into phospholipids, which also enhances their susceptibility to peroxidation [25]. ALOXs (lipoxygenases) are responsible for the oxygenation of PUFAs, and POR (cytochrome P450 oxidoreductase) supplies the electrons needed for lipid peroxidation [26]. The lipid peroxidation that occurs greatly reduces membrane integrity, leading to eventual ferroptotic cell death [24].

### 2.2. Anti-Ferroptosis

While pro-ferroptotic players mostly involve those related to intracellular iron uptake and membrane lipid peroxidation, anti-ferroptotic players are involved in the cell’s antioxidant functions as well as the prevention of both iron accumulation and lipid peroxidation [5]. The xCT system is composed of two transmembrane proteins, SLC7A11 and SLC3A2 (cystine transporter solute carrier family 7 member 11; cystine transporter solute carrier family 3 member 2), which create a cystine/glutamate antiporter [27]. This allows for the uptake of cystine into the cell, which is a key precursor for glutathione (GSH) synthesis. GSH is a key antioxidant defense that aids in the prevention of oxidative stress and lipid peroxidation. GSH is oxidized via glutathione peroxidase 4 (GPX4) to form oxidized GSSG in order to neutralize harmful lipid peroxides, and thus protect the cell from membrane damage and cell death [28]. Iron chelators can bind to and reduce the iron levels in the cell, and ferritin, an iron storage protein, can also contribute to the regulation of intracellular iron levels, preventing ferroptosis [29].

Several other factors and proteins can also influence the increase or decrease in these key pro- or anti-ferroptotic players, which will either promote or inhibit this cell death process.

## 3. Ferroptosis Activation or Inhibition Contributes to Cancer Suppression or Formation

Innately, ferroptosis is a mechanism for tumor suppression, which leads to the cell death of cancerous cells [6]. Mechanistically, this is owed to the interaction with various tumor-suppressor genes such as tumor protein 53 (TP53). The role of tumor suppressors and their interaction with ferroptosis pathways demonstrates that ferroptosis can serve as a natural defense against cancer development [30].

TP53 is the most frequently mutated tumor-suppressor gene and can play an important role in the promotion of ferroptosis in cancer [30]. It is well established that p53 is crucial for restricting tumor cell growth and, in terms of ferroptosis, downregulates SLC7A11 expression, a key component of the xCT system [31]. This occurs through its direct binding to the promoter of SLC7A11 and its interaction with ubiquitin-specific peptidase 7 to reduce histone H2B monoubiquitination on the promoter [32]. p53 also regulates the expression of several other genes involved in cellular metabolism that would enhance ferroptosis in tumor cells, including ferredoxin and vitamin K epoxide reductase complex subunit 1-like 1. Both proteins play key roles in protecting the cell against ferroptosis; therefore, manipulating their activity via p53 can lead to induction of cell death [30]. Acetylation-defective p53 mutant that fails to induce apoptosis in cells induces ferroptosis and leads to tumor suppression [33].

Other tumor-suppressor proteins, BRCA1-associated protein-1 (BAP1) and APC membrane recruitment protein 1 (AMER1), can also contribute to ferroptosis modulation [30]. BAP1 can remove the H2A ubiquitination on the SLC7A11 promoter, therefore decreasing its transcription and reducing intracellular cystine uptake and promoting ferroptosis [34]. AMER1 interacts with SLC7A11 and ferritin light chain, which can promote their degradation, thus promoting ferroptosis [35].

Nuclear factor (erythroid-derived 2)-like 2 (NRF2) is controlled by tumor suppressors kelch-like ECH-associated protein 1 (KEAP1) and alternative reading frame (ARF) [30]. KEAP1 is responsible for targeting NRF2 for degradation, and ARF can inhibit the transcriptional activity of NRF2. When both tumor suppressors are missing, NRF2 is active and can protect against ferroptosis through the regulation of several genes involved in ferroptosis defenses and iron metabolism [36]. Thus, suppression of NRF2 via KEAP1 and ARF is a key tumor suppression mechanism via ferroptosis promotion [30].

Ferroptosis suppressor protein 1 (FSP1) is another molecule found to play a significant role in cancer-related ferroptosis. The targeting of FSP1 allows for the disruption of CoQ10-mediated antioxidant defenses, which is independent of GPX4 activity. This was studied in lung cancer and found to sensitize the cells to ferroptotic cell death. The researchers identified FSP1 as a critical and relevant ferroptosis target, as it expands beyond the GPX4 pathway, allowing for more therapeutic targets [37]. Similarly, Xavier et al. conducted a similar study providing insight into ferroptosis regulation by characterizing iFSP1-like compounds that inhibit AIFM2/FSP1 activity. The authors of this paper explore FSP1 inhibition and demonstrate how disruption of the FSP1/CoQ10 antioxidant pathway sensitizes cells to lipid peroxidation and thus ferroptotic cell death [38]. These studies identified FSP1 as a critical and relevant ferroptosis target, as it expands beyond the GPX4 pathway, allowing for more therapeutic targets.

Another example of ferroptosis promotion involves a study that evaluated cyclin-dependent kinase inhibitor p27 in ovarian cancer. p27 promotes lipid peroxidation and iron-dependent oxidative stress, which was seen to lower the threshold for ferroptosis in response to chemotherapy [39]. As p27 is involved in cell cycle regulation, it can also be used to further establish a link between the cell cycle and ferroptosis and may serve as a potential biomarker or therapeutic target.

Some non-canonical tumor suppressors, like lysine methyltransferase 2B (MLL4), have also been shown to play a role in ferroptosis-mediated tumor suppression, as well. MLL4 is often mutated in human cancers, and its deficiency can result in decreased expression of pro-ferroptosis genes, including ALOX12, ALOX12B, and ALOXE3, as well as increased expression of anti-ferroptosis proteins such as SLC7A11 and GPX4 [30].

Alternatively, several oncogenes have been shown to lead to ferroptosis resistance in cancer cells by promoting the antioxidant defenses that suppress lipid peroxidation and prevent the generation of a labile iron pool and production of ROS—thus allowing for the survival of malignant cells [11].

Mutations in specific oncogenes can also be advantageous to cancer cells in evading ferroptosis. RAS proto-oncogenes play a key role in promoting ferroptosis evasion in cancer cells through SLC7A11-mediated cystine uptake. Cancer cells with mutated RAS have been shown to have increased SLC7A11 transcription, which leads to increased levels of intracellular cysteine and GSH [40].

Some p53 mutations, such as p53R273H, have also demonstrated the ability to upregulate SLC7A11 expression and therefore prevent ferroptosis [30].

Glycolytic enzyme enolase 1 (ENO1) is involved in ferroptosis prevention via its interaction with the iron regulatory protein 1-mitoferrin-1 pathway, which plays a role in iron metabolism. This process leads to the constraint of the labile iron pool, thus suppressing ferroptosis [41].

Tumor cells evade ferroptosis by lowering mitochondrial ROS production. Proteins such as dynamin-related protein 1 (DRP1) interact with mitochondrial cyclic GMP-AMP synthase (cGAS), which leads to the promotion of DRP1-mediated mitophagy and reduces mitochondrial ROS production and thus ferroptosis [42].

Taken together, several different tumor-suppressor genes and oncogenes have been shown to influence ferroptosis in cancer cells. These genes manipulate key players of the ferroptosis pathways that either allow for the induction or inhibition of this form of cell death [43].

## 4. Therapeutic Strategies Targeting Ferroptosis in Cancer

The manipulation of ferroptosis is an attractive target for cancer therapeutics [6]. Current strategies aim to induce or inhibit this cell death mechanism to either eliminate cancer cells or protect normal cells, respectively [1]. Strategies include small-molecule inhibitors, gene-editing technology, and combination therapies [6]. The therapeutic strategy is dependent upon the tumor type, stage, and microenvironment (TME) [6].

The most common therapy to instigate this form of cell death is the induction of ferroptosis, utilizing several different compounds. This is an invaluable cancer therapeutic tool, as several cancer types that are typically resistant to other forms of cell death, such as apoptosis, are found to be susceptible to ferroptosis. For example, erastin and RSL3 are two molecules that have been extensively studied in the induction of ferroptosis [44]. Erastin acts through the inhibition of the xCT antiporter (or Xc^−^ system) composed of both SLC7A11 and SLC3A2. Inhibition of this antiporter leads to decreased intracellular cystine uptake and subsequent depletion of GSH. A reduction in GSH levels leads to weakened GPX4 activity, the accumulation of lipid peroxides, and ferroptosis induction [45]. RSL3 is another induction molecule that directly inhibits GPX4 by bypassing the need for GSH depletion [46]. Although they work through the inhibition of different proteins, both molecules lead to overwhelming oxidative stress and lipid peroxidation and thus, cancer cell death [47].

The targeting of iron metabolism is another area of focus for ferroptosis therapeutics. Some nanoparticles have been developed that contain erastin or iron analogs, which lead to a selective increase in the intracellular labile iron pool and ROS levels within the tumor cells, thus contributing to increased cell death [48].

A well-known chemotherapeutic agent, sorafenib, has also demonstrated ferroptosis induction. An HCC study has shown that sorafenib enhances ferroptosis through the inhibition of SLC7A11 and subsequent decrease in cystine uptake. Combination therapies, including radiotherapy and immune checkpoint blockade, have also been reported to sensitize tumors to ferroptosis in similar ways [49].

When looking at traditional cancer treatments and ferroptosis, Yu et al. were able to identify ferroptosis as an underlying mechanism for radiation-induced mucositis—a dose-limiting toxicity that can occur with radiotherapy. Their study demonstrates that metabolic support can preserve redox homeostasis and lipid integrity in nonmalignant mucosal tissue, which suppresses ferroptotic cell death in normal cells following radiation exposure. This would support a more selective ferroptosis modulation in oncogenic therapies to preserve healthy, noncancerous tissue while targeting malignant cells [50].

More recently, a potent inhibitor of ferroptosis was identified to undermine redox-based cancer therapies. Microbial metabolite indole-3-aldehyde (I3A) was found to activate the aryl hydrocarbon receptor (AHR), which suppresses JNK/c-JUN signaling under oxidative stress. This leads to reduced autophagic flux and stabilization of GPX4, thus decreasing lipid peroxidation. Mouse melanoma models demonstrated reduced antitumor efficacy of RSL3 treatment when I3A was also used. The authors of this article also found that I3A levels were increased in mice with *Lactobacillus reuteri* gut colonization, which was able to confer the mice with ferroptosis resistance. This highlights a potential host–microbiome metabolic axis that should be considered for future therapeutics [51].

## 5. Ferroptosis and Its Effects on the Immune System and Immune Evasion

Many therapies promote ferroptosis within tumor cells and lead to their destruction and elimination; however, in some cases, ferroptosis inhibition may be of therapeutic benefit [52]. Many tumors demonstrate immune evasion and increased aggressiveness when exposed to ferroptosis-related damage-associated molecular patterns (DAMPs), which highlights the context-dependent immune consequences of ferroptotic cell death [12]. In these cases, agents such as ferrostatin-1, liproxstatin-1, and vitamin E analogs have been used to limit both oxidative damage and immune evasion [53].

Immune cells within the tumor microenvironment can also undergo unwanted ferroptosis, impairing their anti-tumor effects. Dendritic cells failed to effectively present tumor antigens as they undergo ferroptosis, resulting in weakened CD8+ T cell responses [54]. The decrease in the effectiveness of these immune cell populations within the TME can affect certain therapeutics, such as immune checkpoint blockade and adoptive T cell therapies. Therefore, inhibiting ferroptosis in these cell populations may increase the efficacy of these treatments [17]. These vulnerabilities can be explained, for example, by lowered GPX4 and FSP1 expression in certain immune subsets, making them more susceptible to lipid peroxidation and ferroptosis than malignant cells [37,38]. Therefore, selective inhibition of ferroptosis in immune cell populations may enhance therapeutic outcomes.

As ferroptosis has been shown to influence tumor cell survival both positively and negatively, it also plays an essential role in shaping the tumor microenvironment [55]. The release of DAMPs and lipid peroxidation products during ferroptotic cell death can also act as immunogenic signals that may lead to the activation of dendritic cells (DCs) and macrophages. These activated APCs can present these tumor-associated antigens to T cells to facilitate their recruitment and activation to enhance antitumor immunity [56]. Oxidized phospholipids, such as cell membrane component phosphatidylethanolamine, and lipid aldehydes, such as 4-hydroxynoneal and malondialdehyde, can be recognized by pattern recognition receptors (PRRs). Important PRRs, including TLR4 and cGAS-STING, are capable of recognizing these phospholipids and, when activated, trigger NF-KB and IRF3 signaling to promote proinflammatory cytokine release and effector cell recruitment [57]. 

Previous studies have also demonstrated that interferon-γ (IFN-γ) plays a key role in ferroptosis and antitumor immunity. IFN-γ, which is secreted by CD8+ T cells, can downregulate the expression of the Xc^−^ system (which includes SLC7A11) in tumor cells, making them more susceptible to ferroptosis. This further enables immune cells to directly kill tumor cells and concurrently makes them much more susceptible to ferroptotic cell death [58].

Under certain conditions, however, ferroptosis can also lead to immune evasion. Excessive lipid peroxidation products, such as the oxidized phospholipids, can impair the viability and survival of immune effector cells, as discussed earlier [59]. In addition to DCs, T cells and natural killer cells (NK cells) within the TME can be affected. Reduced effective antigen presentation and cytotoxic abilities allow for less immune cell involvement to effectively target tumor cells [53]. Tumor cells are able to exploit this by inducing ferroptosis in the surrounding immune cells of the TME by secreting ROS or modulating iron metabolism [60]. Metabolic factors such as glutathione depletion, NADPH availability, and intracellular cystine uptake efficiency can also modulate ferroptosis sensitivity in both tumor and immune cell populations [61].

The balance between ferroptosis in tumor cells and immune cells plays an important role in determining therapeutic outcomes. The inhibition of ferroptosis in tumor-infiltrating lymphocytes and immune cells within the TME, while also promoting it within malignant cells, has been proposed as an effective antitumor immune strategy [62]. Combining this manipulation strategy with other immunotherapies, such as checkpoint blockades, can maintain immune cell function while targeting tumor cells with ferroptotic mechanisms [63].

## 6. Limitations and Promising Opportunities in Therapeutic Targeting

Although there is promising potential in targeting ferroptosis for therapeutic intervention in cancer, it currently poses several challenges. Tumor heterogeneity is a key aspect when it comes to cancer treatment; therefore, ferroptosis treatments may exhibit varying levels of sensitivity in different tumors [64]. Some cancers may develop resistance to therapeutic intervention through pathways such as metabolic reprogramming and/or upregulation of anti-ferroptotic-related molecules [65]. As ferroptosis becomes increasingly studied in the cancer field, many of the unknowns surrounding its therapeutic manipulation may be unveiled.

Currently, emerging tools such as CRISPR gene editing, lipidomics, and single-cell ferroptosis assays are being used to enhance our understanding and uncover more ferroptotic vulnerabilities in different tumor types [66]. Biomarker discovery is also of great interest, as it can help identify ferroptosis sensitivity. ACSL4 has already been identified as such a biomarker. Other genes of interest may include iron-handling genes and even lipid profiles to make way for more personalized therapeutic interventions [10].

Currently, translation of research to the clinic is being explored, but it is very limited. A Phase I/II trial showed promise with an erastin analog (PRLX93936); however, nothing has entered late-stage oncology trials or regulatory approval, as there were tolerance issues in patients [67]. A recent study involving Carbon Nanoparticle-Loaded Iron (CNSI-Fe(II)) was also conducted, and it was hypothesized that this nanoparticle would increase iron overload leading to ferroptosis induction; however, no results have been formally published yet [68]. Trials involving inhibitors of key ferroptosis-related proteins such as GPX4 and the xCT system are also being explored but are still quite preliminary. A considerable amount of clinical activity surrounding ferroptosis centers on combination therapies or the use of already approved cancer therapeutics, such as sorafenib, which underscores the importance of ferroptosis research [69].

## 7. Conclusions

Ferroptosis is a regulated form of cell death that has become highly relevant in the field of cancer studies, particularly because some cancers appear to be resistant to apoptosis while remaining susceptible to ferroptotic death. Its role in cancer is context-dependent, as it functions as both a tumor suppressor and tumor promoter, depending on cellular state and the TME. This “double-edged sword” nature arises from tightly regulated interactions among iron metabolism, lipid peroxidation, and redox balance—all of which are influenced by oncogenes, tumor-suppressor genes, and environmental cues within the TME. Those discussed in this paper are summarized in Table 1.

Therapeutically, ferroptosis presents as both promising and challenging. Small molecules such as erastin, RSL3, and sorafenib can induce ferroptosis in malignant cells, with enhanced efficacy when combined with modalities such as preexisting immunotherapies. However, excessive or poorly targeted ferroptosis may impair immune function and promote immune evasion, which underscores the need for precise targeting and modulation. Ongoing challenges include tumor heterogeneity, adaptive resistance, and an incomplete understanding of ferroptosis regulation across different cancer types. Advances in technologies such as CRISPR, lipidomics, and ferroptosis-specific assays are all expected to aid in the identification of novel biomarkers and therapeutic targets, enabling more personalized and effective ferroptosis-based cancer treatments.

## Figures and Tables

**Table 1:** Summary of the players involved in both pro-ferroptotic and anti-ferroptotic processes.

Category	Player	Function/Mechanism	Effect on Ferroptosis	References
Iron metabolism	Transferrin receptor	Mediates uptake of ferric iron (Fe^3+^) into the cell	Promotes ferroptosis by increasing the intracellular iron pool	[17]
Iron metabolism	STEAP3	Reduces Fe^3+^ to Fe^2+^ in endosomes	Increases labile Fe^2+^ available for the Fenton reaction	[17]
Iron metabolism	NCOA4	Promotes ferritinophagy (ferritin degradation)	Increases labile iron pool and ROS generation	[19]
Iron metabolism	Ferritin	Stores iron and prevents free iron accumulation	Inhibits ferroptosis by limiting iron availability	[19,26,32]
Iron metabolism	Iron chelators	Binds and sequesters free iron	Inhibits ferroptosis	[26]
Lipid Metabolism	ACSL4	Activates PUFAs for incorporation into phospholipids	Promotes lipid peroxidation	[21,22,66,70]
Lipid Metabolism	LPAT3	Incorporates PUFAs into membrane phospholipids	Enhances susceptibility to lipid peroxidation	[21,22,71]
Lipid Metabolism	ALOXs	Catalyzes oxygenation of PUFAs to lipid peroxides	Promotes ferroptosis	[21,23,27]
Lipid Metabolism	POR (cytochrome P450 oxidoreductase)	Supplies electrons for lipid peroxidation	Promotes ferroptosis	[23,72]
Antioxidant Defense	xCT System (SLC7A11/SLC3A2)	Imports cystine for glutathione (GSH) synthesis	Inhibits ferroptosis by sustaining antioxidant defense	[24,28,39,51,73]
Antioxidant Defense	Glutathione (GSH)	Major antioxidant reducing lipid peroxides	Inhibits ferroptosis	[25,74]
Antioxidant Defense	GPX4	Helps neutralize harmful lipid peroxides	Inhibits ferroptosis	[25,75]
Tumor Suppressors and Oncogenes	TP53	Repress SLC7A11 transcription and promote ROS production	Promote ferroptosis	[27,75]
Tumor Suppressors and Oncogenes	BAP1	Deubiquitinate histone H2A on SLC7A11 promoter	Promote ferroptosis	[27,31]
Tumor Suppressors and Oncogenes	AMER1	Promote degradation of SLC7A11 and ferritin	Promote ferroptosis	[27,32]
Tumor Suppressors and Oncogenes	NRF2	Activate antioxidant and iron metabolism genes	Inhibit ferroptosis	[27,33,76]
Tumor Suppressors and Oncogenes	KEAP1/ARF	Negatively regulate NRF2	Promote ferroptosis	[27,33,76]
Tumor Suppressors and Oncogenes	MLL4	Regulate expression of ferroptosis-related genes	Promote ferroptosis	[27,77]
Tumor Suppressors and Oncogenes	RAS (mutant)	Upregulate SLC7A11, which in turn increases GSH	Inhibit ferroptosis	[34]
Tumor Suppressors and Oncogenes	ENO1	Restrict labile pool of iron via the iron regulatory protein 1-mitoferrin-1 pathway	Inhibit ferroptosis	[35,78]
Tumor Suppressors and Oncogenes	DRP1/cGAS	Enhance mitophagy, which reduces mitochondrial ROS production	Inhibit ferroptosis	[36]

## Data Availability

No new data were created or analyzed in this study. Data sharing is not applicable to this article.

## Abbreviations

The following abbreviations are used in this manuscript:

ROS	Reactive oxygen species
PUFA	Polyunsaturated fatty acid
HCC	Hepatocellular carcinoma
STEAP3	Six-transmembrane epithelial antigen of prostate 3
NCOA4	Nuclear receptor coactivator 4
ACSL4	Acyl-CoA synthetase long-chain family member 4
LPAT3	Lysophosphatidylcholine acyltransferase 3
ALOX	Arachidonate lipoxygenase
POR	Cytochrome P450 oxidoreductase
xCT or Xc^−^	Cystine/glutamate antiporter system
SLC7A11	Cystine transporter solute carrier family 7 member 11
SLC3A2	Cystine transporter solute carrier family 3 member 2
GSH	Glutathione
GPX4	Glutathione peroxidase 4
GSSG	Glutathione disulfide
TP53	Tumor protein 53 gene
p53	Tumor protein 53
BAP-AMER1	BRCA-1 associated protein-1
NRF2	APC membrane recruitment protein 1
KEAP1	Nuclear factor (erythroid-derived 2)-like 2
ARF	Kelch-like ECH-associated protein 1
FSP1	Alternative reading frame
MLL4	Ferroptosis suppressor protein 1
RAS	Lysine methyltransferase 2B
ENO1	Rat sarcoma
DRP1	Enolase 1
cGAs	Dynamin-related protein 1
TME	Mitochondrial cyclic GMP-AMP synthase
RSL3	Tumor microenvironment
DAMPs	RAS-selective lethal 3
DCs	Damage-associated molecular patterns
APC	Dendritic cells
PRR	Antigen-presenting cell
IFN-γ	Pattern-recognition receptor
NK cells	Interferon gamma
PD-1	Natural killer cells
PD-L1	Programmed cell death protein 1
